# Editorial: Advances in genomic and genetic tools, and their applications for understanding embryonic development and human diseases

**DOI:** 10.3389/fcell.2022.1016400

**Published:** 2022-11-21

**Authors:** Muhammad Abu-Elmagd, Mourad Assidi, Abdulmajeed F. Alrefaei, Ahmed Rebai

**Affiliations:** ^1^ Center of Excellence in Genomic Medicine Research, King Abdulaziz University, Jeddah, Saudi Arabia; ^2^ Department of Medical Laboratory Technology, Faculty of Applied Medical Sciences, King Abdulaziz University, Jeddah, Saudi Arabia; ^3^ Department of Biology, Jamoum University College, Umm Al-Qura University, Mecca, Saudi Arabia; ^4^ Centre of Biotechnology of Sfax, University of Sfax, Sfax, Tunisia

**Keywords:** genetic/genomic tools, gene expression and regulation, embryonic development, human diseases, Bioinformatics tools, Dual function genes, Molecular signaling

## Abstract

Significant advances have been recently made in the development of the genetic and genomic platforms. This has greatly contributed to a better understanding of gene expression and regulation machinery. Consequently, this led to considerable progress in unraveling evidence of the genotype-phenotype correlation between normal/abnormal embryonic development and human disease complexity. For example, advanced genomic tools such as next-generation sequencing, and microarray-based CGH have substantially helped in the identification of gene and copy number variants associated with diseases as well as in the discovery of causal gene mutations. In addition, bioinformatic analysis tools of genome annotation and comparison have greatly aided in data analysis for the interpretation of the genetic variants at the individual level. This has unlocked potential possibilities for real advances toward new therapies in personalized medicine for the targeted treatment of human diseases. However, each of these genomic and bioinformatics tools has its limitations and hence further efforts are required to implement novel approaches to overcome these limitations. It could be possible that the use of more than one platform for genotype-phenotype deep analysis is an effective approach to disentangling the cause and treatment of the disease complexities. Our research topic aimed at deciphering these complexities by shedding some light on the recent applications of the basic and advanced genetic/genomic and bioinformatics approaches. These include studying gene-gene, protein-protein, and gene-environment interactions. We, in addition, aimed at a better understanding of the link between normal/abnormal embryonic development and the cause of human disease induction.

## Introduction

The current Research Topic (RT) was aimed at deciphering the molecular signaling pathways’ interactions that orchestrate the complexity of embryonic development and human disease. Understanding these pathways should aid in developing targeted therapies. Several reports showed that many genes and/or microRNAs have a dual functional role during normal embryonic development and in human disease conditions (Miyaki et al., 2010; Ulveling et al., 2011; Hosseini-Farahabadi et al., 2013; Lan et al., 2019; Mahajan et al., 2021). Hence, discovering the missing links driving changes in gene expression and regulation during embryonic development to cause human disease induction has been a hot topic for many years (Loch-Caruso and Trosko, 1985; Nunes et al., 2003; Taylor et al., 2004; Lees et al., 2005; Kloosterman and Plasterk, 2006; Hashimoto et al., 2014; Degrauwe et al., 2016; Mazzoccoli et al., 2016; Tatetsu et al., 2016; Shah et al., 2018; Chahal et al., 2019; Prieto-Colomina et al., 2021). The themes of the RT attracted 61 research groups around the world to submit their work among which 32 manuscripts were peer-reviewed and accepted https://www.frontiersin.org/research-topics/10510/advances-in-genomic-and-genetic-tools-and-their-applications-for-understanding-embryonic-development. These included 26 original research articles, 3 case reports, 1 brief research report, 1 systematic review, and 1 opinion article (Table 1). The findings of these articles have certainly added to our knowledge and understanding of the field and have widely opened the door for more extended research to be carried out by other research groups worldwide. Without a doubt, several accepted manuscripts have achieved the main goals of the Research Topic. The main findings of these articles were classified into 3 main research themes and highlighted as follows:

**TABLE 1:** A list of the accepted articles of the research topic summarizing the research area of each study, article’s title, article’s type, the model used, tool/platform used, main findings and the reference to each study.

No.	Area of Research	Title of the Article	Type of the Article	Model Used	Tools/Platforms Used	Main Findings	Authors
1 Unraveling Diseases’ Complexity Using Embryonic and In Vitro Model Systems							
1	Mimicking human diseases in embryonic model systems	SLC20A1 is Involved in Urinary Tract and Urorectal Development.	Original Research	-Zebrafish embryos.	-Morpholino oligonucleotide knockdown.	-SLC20A1 gene is involved in urinary tract and urorectal development.	Rieke et al. (2020).
				-Human patients.	-Western Blotting.	-Human SLC20A1 is implicated as a disease gene for bladder exstrophy-epispadias complex.	
					-Sanger sequencing.		
					-PCR		
					-Immunohistochemistry.		
2		Starvation to Glucose Reprograms Development of Neurovascular Unit in Embryonic Retinal Cells.		- Mouse embryonic retinal cells.	-In vitro retinal cell cultures.	-Glucose deprivation during early embryonic development could differentially alter the expression of genes in the retinal neurovascular unit.	Özgümüs et al. (2021)
					-RNASeq.	-Abnormal embryonic programming during retinogenesis might lead to severe retinopathy in patients with diabetes.	
					-Gene ontology and pathway enrichment Analyses.		
					-Immunohistochemistry staining.		
3		Placenta-Derived Osteoprotegerin is Required for Glucose Homeostasis in Gestational Diabetes Mellitus.		-Maternal mice.	-*In vitro* tissue cultures.	-Placenta-derived Osteoprotegerin (OPG) regulated glucose homeostasis during pregnancy via enhancement of β- cell proliferation.	Huang et al. (2020).
				-Blastocysts transduced with lentiviruses to establish placenta- specific OPG knockdown or overexpression mouse models.	-ELISA	-A novel feedback mechanism related to the regulation of glucose homeostasis during pregnancy.	
				-Diabetes mellitus patients’ serum.	-Western Blotting.		
					-Intraperitoneal glucose tolerance tests (IPGTTs).		
					-Immunofluorescence staining.		
4		Single-Cell Transcriptomic Analysis Reveals Mitochondrial Dynamics in Oocytes of Patients with Polycystic Ovary Syndrome.	Original Research	- Data analysis of oocytes from patients with polycystic ovary syndrome (PCOS) at the germinal vesicle, metaphase I, and metaphase II stages versus healthy fertile women.	-Freely available data collection.	- Mitochondrial function at the germinal vesicle stage may contribute to a decline in oocyte quality in PCOS patients.	Qi et al. (2020).
					-Weighted gene co- expression network Analysis (WGCNA).		
					-Gene clustering and differential expression analysis.		
					-Gene ontology and KEGG analysis.		
5	Studying Embryonic Gene in Diseases	Up-regulation of SALL4 Is Associated with Survival and Progression via Putative WNT Pathway in Gastric Cancer.		- Gastric cancer (GC) patients.	-TCGA & gene expression Omnibus data retrieval.	-SALL4 is associated with clinicopathological features related to cancer progression in GC and its function in the Wnt/b- catenin pathway.	Yang et al. (2021).
					-Gene set enrichment analysis.	-SALL4 could be served as a marker for prognostic prediction in GC.	
					-Weighted gene co- expression network analysis.		
6	Human-Derived Stem Cells-Based Therapy Approaches	Deciphering the Association of Cytokines, Chemokines, and Growth Factors in Chondrogenic Differentiation of Human Bone Marrow Mesenchymal Stem Cells Using an ex vivo Osteochondral Culture System.		- Bone marrow mesenchymal cells (BM-MSCs) were collected from Osteoarthritis (OA) patients.	-Tissue culture of BM- MSCs.	-Detected differential secretion of several growth factors, chemokines, pro-inflammatory cytokines, and anti-inflammatory cytokines in the experimental groups compared to the control.	Jafri et al. (2019).
					-Multiplex Luminex xMAP Assay of chemokines, pro- inflammatory and anti- inflammatory		
					Cytokines, growth factors.	-Stem cells and local cartilage tissues could be used for cartilage differentiation and the management of OA.	
					-Ingenuity Pathway Analysis (IPA).		
7		In vitro Evaluation of the Anti-inflammatory Effects of Thymoquinone in Osteoarthritis and in silico Analysis of Inter- Related Pathways in Age-Related Degenerative Diseases.		- Bone marrow mesenchymal cells (BM-MSCs) were collected from Osteoarthritis (OA) patients.	- Cell Metabolic Activity (MTT assay), Flow cytometry analysis of CD Markers, CellTiter-BlueR assay, Adipocytic differentiation potential of BM-MSCs, Gene expression profiling (qRT- PCR), Bioinformatics assays: Ingenuity Pathway Analysis (IPA) & SwissTargetPrediction.	-Thymoquinone (TQ) reduced cell viability, downregulated pro- apoptotic BAX gene, and upregulated anti-inflammatory & Survivin genes.	Kalamegam et al. (2020).
						-Pathways related to synaptogenesis, neuroinflammation, TGF-b & interleukin signaling could be affected.	
8		Human Wharton’s Jelly Stem Cell Secretions Inhibit Human Leukemic Cell Line K562 in vitro by Inducing Cell Cycle Arrest and Apoptosis.		Human umbilical cords (hUCs) to isolate human Wharton’s jelly stem cells (hWJSCs)	-Cell metabolic activity MTT assay, Cell cycle Propidium iodide assay, cytokine analysis using multiplex bead-based immunoassay, cell death Annexin V–APC & PI assay.	-hWJSCs/hWJSC extracts decreased the leukemic cell number by inducing cell cycle arrest & apoptosis.	Huwaikem et al. (2021).
					-qRT-PCR gene expression assay.	-hWJSCs/hWJSC extracts upregulated anti-inflammatory cytokines & downregulated pro-inflammatory genes & cytokines that might lead to the inhibition of the K562 cells.	
2 Applications of Advanced Genomics & Bioinformatics Tools in Diseases							
2.1 Applications of Advanced Genomics Tools in Diseases:							
9	Genomic Tools Applications (RNA-Seq, WES, mtDNA-Seq, eQTL.	Combined Analysis of Expression Profiles in a Mouse Model and Patients Identified BHMT2 as a New Regulator of Lipid Metabolism in Metabolic-Associated Fatty Liver Disease.	Original Research	-Farnesoid X receptor (FXR) knockout (KO) mice.	-Hepatic transcriptome including RNA-seq of the overlapped differentially expressed genes in the FXR KO mice and MAFLD patients.	- BHMT2 and PKLR could be highly correlated with MAFLD.	Ma et al. (2021).
				-Patients with metabolic- associated fatty liver disease (MAFLD).	-Correlation analysis, hierarchical clustering, gene ontology (GO) & pathway analysis.		
10		Expression Quantitative Trait Loci (eQTL) Mapping in Korean Patients with Crohn’s Disease and Identification of Potential Causal Genes Through Integration with Disease Associations.		-Korean CD patients' whole-blood	-Whole-blood RNA- sequencing.	Identifying new eQTL associated with Crohn’s Disease.	Jung et al. (2020).
					-Expression quantitative trait loci (eQTL).		
					-Enrichment analysis on eGene Set.		
11		Genome-Wide Association of Proprotein Convertase Subtilisin/Kexin Type 9 Plasma Levels in the ELSA-Brasil Study.		-Brazilian subjects enrolled in the Estudo Longitudinal de Saude do Adulto cohort.	-Axiom_PMRA.r3 array.	- PCSK9 levels may be modulated by trans genetic variation outside of the PCSK9 gene.	Bensenor et al. (2021).
					-Used TOPMED multi- ancestry sample panel as a reference.		
					-Genome-wide association, SNP genotyping.		
					-LocusFocus analytical approach for colocalization testing.		
					-ELISA.		
12		MDR1 Gene Polymorphisms and its Association with Expression as a Clinical Relevance in Terms of Response to Chemotherapy and Prognosis in Ovarian Cancer.		-Patients with ovarian cancer/Biopsies/blood samples.	-Exome sequencing	- Exons 12 (C1236T) & exon 26 (C3435T) polymorphism may play a role in drug resistance by altering the expression level of MDR1 gene.	Haque et al. (2020).
					-RT-qPCR		
					-Restriction fragment length polymorphism (RFLP) analysis.		
13		Clinical Utility of Rapid Exome Sequencing Combined with Mitochondrial DNA Sequencing in Critically Ill Pediatric Patients with Suspected Genetic Disorders.		- Pediatric patients with suspected genetic disorders such as neuromuscular or other systemic abnormalities.	- Rapid clinical exome sequencing (CES) combined with mtDNA sequencing.	- Rapid mitochondrial sequencing combined with exome sequencing in patients diagnosed with suspected mitochondrial disorders is an efficient approach to avoid a wide range of unnecessary clinical investigations.	Ouyang et al. (2021).
14		Exome Analysis Identified Novel Homozygous Splice Site Donor Alteration in NT5C2 Gene in a Saudi Family Associated with Spastic Diplegia Cerebral Palsy, Developmental Delay, and Intellectual Disability.		-Human subjects with neurodevelopmental disorders.	-Electroencephalography.	- Identified a homozygous splice site donor alteration of possible interest in NT5C2.	Naseer et al. (2020b).
					-Whole exome sequencing (WES).		
					-Sanger sequencing.		
					-PCR		
15	Targeted exome Seq for diagnosis	Genetics Evaluation of Targeted Exome Sequencing in 223 Chinese Probands with Genetic Skeletal Dysplasias.		- Children and young adults.	-Sanger sequencing.	- Targeted exome sequencing (TES) achieved a high diagnostic rate for Genetic Skeletal Dysplasias (GSD) which led to better & Improved clinical management.	Lv et al. (2021).
					-Targeted Exome. Sequencing (TES) & variant analysis.		
					-Cost-efficiency analysis.		
16	Identifying Gene Variants	Novel Missense Variant in Heterozygous State in the BRPF1 Gene Leading to Intellectual Developmental Disorder with Dysmorphic Facies and Ptosis.	Brief Research Report	- Human subjects.	-Whole Exome Sequencing (WES).	- BRPF1 gene variants is associated with an intellectual developmental disorder with dysmorphic facies and ptosis.	Naseer et al. (2020a).
					-Sanger sequencing.		
					-PCR validation.		
					-Computational structural analysis of the		
					mutants.		
17		Genetic Variants and Functional Analyses of the ATG16L1 Gene Promoter in Acute Myocardial Infarction.	Original Research	-Acute myocardial- infarction (AMI) patients.	-DNA/PCR sequencing.	- Identified 10 SNPs, 2 DNA- variants, & 3 ATG16L1 gene promoter mutations in AMI patients. These mutations affect the binding of transcription factors and change the transcriptional activity of ATG16L1.	Han et al. (2021).
				-HEH2, HEK-293, and H9c2 cells.	-Dual-luciferase reporter assay.		
					-Nuclear extracts/ electrophoretic mobility shift assay.		
18		Relationship Between the ApaI (rs7975232), BsmI (rs1544410), FokI (rs2228570), and TaqI (rs731236) Variants in the Vitamin D Receptor Gene and Urolithiasis Susceptibility: An Updated Meta Analysis and Trial Sequential Analysis.	Systematic Review	- Meta-analysis study, data were extracted from different databases.	-Literature search.	- Vitamin-D Receptor (VDR) variants were correlated with urolithiasis susceptibility.	Chen et al. (2020).
					-Data extraction.		
					-Literature quality Evaluation.		
					- Trial sequential analysis (TSA).	- T-allele might be the risk gene and the protective gene in VDR TaqI variant.	
19		Identification and Potential Clinical Utility of the MTNR1B rs10830963 Core Gene Variant Associated to Endophenotypes in Gestational Diabetes Mellitus.	Opinion	- Published literature.	- Published data analysis to find out MTNR1B rs10830963 association with endophenotypes in GDM.	-MTNR1B rs10830963 risk variant is associated with major forms of clinical consequences mainly early prevention failure in high-risk pregnancy, therapy in high-risk Gestational Diabetes Mellitus (GDM) and a neonatal complication-related trait.	Firneisz et al. (2020).
						-Maternal genome wide association studies GWAS on neonatal birthweight may potentially expect the reduction of complications.	
20	Identifying CNVs	Copy Number Variations Analysis Identifies QPRT as a Candidate Gene Associated With Susceptibility for Solitary Functioning Kidney.	Original Research	- Fetuses clinically diagnosed with a solitary functioning kidney.	-Chromosomal microarray analysis (CMA).	-Identified a total of 45 CNVs. CNV 16p11.2 was the highest record with kidney anomalies.	Zhou et al. (2021).
					-qRT-PCR.	-QPRT was distinctly localized in renal tubules.	
					-QPRT siRNA	-Loss of QPRT affected the cell cycle & proliferation of human embryonic kidney cells.	
					knockdown.		
					-Immunohistochemistry staining.		
21	Identifying Mutations	Two Novel Compound Heterozygous Mutations in the TRAPPC9 Gene Reveal a Connection of Non-syndromic Intellectual Disability and Autism Spectrum Disorder.	Case Report	- Human subjects.	-Target enrichment (TruSight One Expanded).	- Two new missense mutations in the TRAPPC9 gene in one individual with intellectual disability (ID) and autism spectrum disorder (ASD).	Krämer et al. (2020).
					-NGS of the coding areas of interest & sequences analysis with SeqNext (JSI).		
					-Sanger sequencing and segregation analysis for validation.		
22		An APC Mutation in a Large Chinese Kindred with Familial Adenomatous Polyposis Was Identified Using Both Next Generation Sequencing and Simple STR Marker Haplotypes.		- Human subjects.	-Next-generation sequencing (NGS).	- APC: p.W553X mutation was detected in all Familial Adenomatous Polyposis studied cases suggesting a potential role in detecting the disease.	Zhan et al. (2020).
					-Targeted sequencing.		
					-PCR-based microsatellite analysis of the APC gene.		
23	Mutation Repair	CRISPR/Cas9-Mediated in vivo Genetic Correction in a Mouse Model of Hemophilia A.	Original Research	- Hemophilia A (HA) mouse model.	-Generated Hemophilia A mouse model.	- Potential treatment of the recurrent mutation in HA patients using *in vivo* gene repair strategy.	Luo et al. (2021).
					-RT-qPCR		
					-CRISPR design & injection.		
					-Next Generation Sequencing (NGS).		
					-Immunofluorescence staining.		
24	Identifying Deletions	Case Report: Candidate Genes Associated with Prenatal Ultrasound Anomalies in a Fetus with Prenatally Detected 1q23.3q31.2 Deletion.	Case Report	- Human subjects.	-Ultrasound examination.	-PBX1 could be a candidate gene for fetal growth restriction, renal hypoplasia and congenital heart disease.	Song et al. (2021).
					-Chromosomal. microarray analysis.	-Correlating the genotype- phenotype might improve prenatal diagnosis of fetuses with chromosome 1q deletion.	
25		Association of CCR5Δ32 Deletion and Human Cytomegalovirus Infection with Colorectal Cancer in Tunisia.		- Blood samples from colorectal cancer patients and healthy subjects.	-Conventional & Nested PCR.	-No association between CCR5Δ32 mutation and colorectal cancer (CRC).	Chelbi et al. (2021).
					-HCMV specific serum IgG and IgM antibodies were investigated by enzyme- linked immunosorbent assay.	-Human cytomegalovirus (HCMV) infection is related to CRC.	
2.2 Applications of Advanced Bioinformatics Tools in Diseases:							
26 71941681263650	Bio i nformati cs	No Casual Relationship Between T2DM and the Risk of Infectious	Original Research	-European ancesty data was obtained from UK Biobank (UKB)	- Two-sample Mendelian randomization (MR).	- No strong evidence to support the causal associations between T2DM and several infectious diseases including Sepsis, skin, Pneumonia, and GUI in the European population.	Wang et al. (2021).
		Diseases: A Two-Sample Mendelian Randomization Study.		The data are of sepsis, skin and soft tissue infections (SSTIs), Urinary tract infections (UTIs), Pneumonia, and genito-urinary infection (GUI) in pregnancy.	- T2DM-related single nucleotide polymorphisms (SNPs).		
27		Decoding the Role of Sphingosine-1-Phosphate in Asthma and Other Respiratory System Diseases Using Next Generation Knowledge Discovery Platforms Coupled with Luminex Multiple Analyte Profiling Technology.		-Human subjects with asthma.	-High throughput next- generation knowledge discovery platforms including: SwissTargetPrediction, WebGestalt, Open Targets Platforms & Ingenuity Pathway Analysis.	- Identified several upstream regulators of Sphingosine-1- phosphate (S1P) signaling including various cytokines and growth factors.	Bahlas et al. (2020).
				-wGSEA gene lists obtained from large-scale-omics studies.	-Multiple analyte Luminex profiling (xMAP).		
28		Screening and Identification of Therapeutic Targets for Pulmonary Arterial Hypertension Through Microarray Technology.		-Data from Gene Expression Omnibus (GEO) database.	- Gene set enrichment analysis (GSEA).	- Identified hub genes and key pathways of pulmonary arterial hypertension (PAH), with a total of 110 differentially expressed genes (DEGs) and 9 hub genes related to PAH.	Li et al. (2020).
				-Microarray data.			
29		Identification of Potential Core Genes Associated with the Progression of Stomach Adenocarcinoma Using Bioinformatic Analysis.		- Stomach adenocarcinoma (STAD) datasets were downloaded from the Gene Expression Omnibus (GEO).	-DEGs discriminated using GEO2R and Venn diagram.	-Upregulated DEGs were enriched in receptor Interaction.	Yang et al. (2020).
					-Gene ontology (GO) and Kyoto Encyclopedia of Gene.	-Downregulated DEGs were involved in gastric acid secretion	
					-Genome (KEGG) enriched pathways to annotate and visualize with DEGs.	-10 core genes exhibited. Significantly higher expression in STAD.	
					-STRING to identify the functional interactions of DEGs.		
					-Selected the most significant DEGs selected to construct the protein-protein interaction (PPI) network.		
					- Kaplan-Meier Plotter and Gene Expression Profiling Interactive Analysis (GEPIA).		
3. Evaluation of the Prognostic Value of Gene Expression in Cancer							
30	Evaluation of the Prognostic Value of Biomarkers in Lung Cancer, Pancreatic Cancer & Leukemia	Evaluation of the Prognostic Value of STEAP1 in Lung Adenocarcinoma and Insights into Its Potential Molecular Pathways via Bioinformatic Analysis.	Original Research	- Clinical data from human subjects was obtained from The Cancer Genome Atlas (TCGA) and the Gene Expression Omnibus (GEO).	-RT-PCR.	-STEAP1 expression is upregulated in Lung Adenocarcinoma (LUAD).	Guo et al. (2020).
					-Western Blotting.	-STEAP1 is associated with the occurrence and progression of LUAD.	
					-Gene ontology (GO) analysis.	-STEAP1 coexpression with other genes could regulate homologous recombination, p53 signaling, cell cycle, DNA replication, and apoptosis.	
					-Gene Analysis using Kyoto Encyclopedia.	-STEAP1 is a potential prognostic biomarker for LUAD.	
					-Genomes (KEGG) analysis.		
					-Gene set enrichment analysis (GSEA).		
31		A Novel DNA Replication-Related Signature Predicting Recurrence After R0 Resection of Pancreatic Ductal Adenocarcinoma: Prognostic Value and Clinical Implications.		-Pancreatic ductal adenocarcinoma (PDAC) human tissue.	-Nomogram.	-7-gene signature was identified.	Feng et al. (2021).
				-Gene expression clinical data collected from ArrayExpress database.	-Univariate Cox regression analysis.	-Signature was closely related to cell cycle, DNA replication, & DNA repair.	
				-RNA-sequencing data and Matched clinical data of TCGA dataset from TCGA hub at UCSC Xena	-Lasso regression analysis.		
					Kaplan-Meier (K-M) survival analysis.		
					-qRT-PCR		
32		Acute Myeloid Leukemia in Qatar (2010–2016): Clinical, Biological, and Prognostic Factors and Treatment Outcomes.		- Patients diagnosed with AML	- Multicolor flow cytometry.	- Applying NGS o AML patients’ diagnoses together with protocols for target therapy could better improve the quality of the patient’s care.	El Omri et al. (2020).
					-Karyotyping		
					-Molecular Mutation Analyses.		
					-Chemotheraphy treatment		

## Unraveling disease complexity using embryonic and *in vitro* model systems

The RT included eight articles under this category in which either a developmental gene was analyzed during embryonic development and in disease, a known developmental gene analyzed in human disease or stem cells were used as an *in vitro* experimental model. In a consortium study, Rieke and his colleagues Rieke et al., showed that SLC20A1 is involved in urinary tract and urorectal development. The study used morpholino to manipulate SLC20A1 function in the zebrafish embryo and the gene was analyzed in patients. The study showed that SLC20A1 was associated with bladder exstrophy-epispadias complex (BEEC, genitourinary malformations). Another research group in the RT used mouse embryonic retinal cells as their model and RNA-seq analysis to study perinatal glucose deprivation during early embryonic development and showed changes in gene expression during retinal neurogenesis. The study concluded that abnormal embryonic programming during retinogenesis could lead to retinopathy in patients with diabetes Özgümüs et al. Mice blastocysts transfected with lentivirus aiming at establishing placenta-specific knockdown or overexpression mouse model were used to study the role of Osteoprotegerin (OPG) in gestational diabetes mellitus (GDM). The study showed that OPG regulates glucose homeostasis during pregnancy through a negative feedback mechanism Huang et al. Polycystic ovary syndrome (PCOS) is another important disease studied in the RT using mitochondrial sequencing of patient oocytes. Transcriptomic dynamics analysis at the single-cell level using the weighted gene co-expression network analysis (WGCNA) approach was applied to show that abnormal mitochondrial function may contribute to a deteriorated oocyte quality in patients diagnosed with polycystic ovary syndrome (PCOS). The authors concluded that the oocyte quality might be affected by mitochondrial function Qi et al. Sall4 is a developmental gene that plays a crucial role in the pluripotency of embryonic stem cells (Chen et al., 2022). In this RT, SALL4 expression was analyzed in gastric cancer patients using gene set enrichment and found to be associated with the cancer progression through Wnt/β-catenin signaling pathway Yang et al. This study reiterated previously reported findings that there is a direct link between development and cancer (Papaioannou et al., 1984; Cheng et al., 2004; Tzukerman et al., 2006; Ma et al., 2010).

Human stem cells are an *in vitro* model system that offers great promise for developmental studies, disease research, and the emergence of targeted therapies. In our RT, mesenchymal stem cells (BM-MSCs) derived from the bone marrow of osteoarthritis (OA) patients were used to show that the expression of several chemokines, and pro- and anti-inflammatory factors were upregulated. The authors concluded that BM-MSCs could be used for cartilage differentiation and repair Jafri et al. Another study showed that Thymoquinone natural product has anti-inflammatory properties in BM-MSCs derived from OA patients suggesting a therapeutic potential of this natural product Kalamegam et al. Moreover, Wharton’s Jelly stem cell secretion was used *in vitro* to suppress human leukemia cells through cell cycle arrest and apoptosis induction Huwaikem et al.

In summary, the research theme discussed above showed that analyzing gene expression and regulation during embryonic development could give clues to human disease induction and progression. Several developmental genes have a functional dual role in development and disease including SLC20A1, Osteoprotegerin, and SALL4. Stem cell-based therapies or regenerative medicine represent a highly promising alternative route for disease cure.

## Applications of advanced genomics and bioinformatics tools in disease

A considerable number of articles in the RT aimed at using a wide range of genomic/genetic and/or bioinformatics tools to decode the biological complexity and identify causal genes in human diseases were published (Table 1). These articles fall, in general, into the following areas:

### Applications of advanced genomic tools in disease

Authors of articles published in this RT applied several advanced genomic tools including RNA-sequencing Jung et al. and Rieke et al., genome-wide association Bensenor et al., and whole exome-sequencing Naseer et al., Haque et al., and Ouyang et al. There was a tendency in several studies for using a combination of more than one genomic tool to critically discover the gene/marker/factor associated with the disease. For example, BHMT2 was identified as a new regulator for lipid metabolism in metabolic-associated fatty liver disease by combining RNA-seq analysis of the expression profile both in a mouse model and human patients Ma et al. In another study, RNA-seq was combined with the eQTL analysis to show the association of causal genes in Crohn’s disease Jung et al. Moreover, a combined exome and mitochondrial sequencing were successfully used for the rapid diagnosis of suspected pediatric mitochondrial disorders Ouyang et al. Next-generation sequencing (NGS) combined with simple short tandem repeat (STR) marker haplotypes were both used to identify a mutation in the adenomatous polyposis coli (APC) of Chinese kindred diagnosed with familial adenomatous polyposis (FAP) Zhan et al.

Targeted exome sequence (TES) was recommended in one of the RT studies as an effective approach for the genetic skeletal dysplasias (GSD) diagnosis Lv et al. Additionally, several studies identified novel genetic variants associated with many disorders and/or diseases such as developmental disorders Naseer et al., acute myocardial infarction Han et al., urolithiasis Chen et al., and endophenotypes in gestational diabetes Firneisz et al. Other RT studies identified copy number variants (CNVs) Zhou et al., gene mutations Krämer et al. and Zhan et al., and gene deletions Chelbi et al. and Song et al., with all, were shown to be associated with different human diseases.

An interesting RT study used a genomic CRISPR/Cas9 mutation repair approach and showed for the first time successful treatment of Hemophilia A in a mouse model Luo et al. This with no doubt gives hope to follow this approach for treatment in humans for Hemophilia A or any other mutation associated with other diseases.

### Applications of advanced bioinformatics tools in disease

There have been significant advances in the use of bioinformatics tools and datasets available online that could be used for deciphering the complexity of human disease (Pereira et al., 2020). In our RT, these freely available online resources were used to decode the functional role of genes in human disease. For example, knowledge-based bioinformatics tools such as SwissTargetPrediction, WebGestalt, Open Targets Platforms and Ingenuity Pathway Analysis (IPA) were used to analyze the role of Sphingosine-1-phosphate in respiratory system diseases Bahlas et al., and to identify therapeutic targets for pulmonary arterial hypertension Li et al. Other tools such as Gene Expression Omnibus (GEO), Gene ontology (GO), Kyoto Encyclopedia of Gene, Genome (KEGG) enriched pathways, and STRING online database were applied to identify the functional interactions of the differentially expressed genes (DEGs) associated with the progression of stomach adenocarcinoma Yang et al.

In summary, the research theme described above focused on identifying causal genes, gene variants, copy number variants, mutations, or deletions associated with human diseases. A wide range of advanced bioinformatics tools was used to analyze the genomic data. Combined genomic/genetic/bioinformatics tools and model systems were applied to decipher the mechanistic complexity of the disease.

## Evaluation of the prognostic value of gene expression

Gene ontology (GO), gene analysis using Kyoto Encyclopedia, genomes (KEGG) analysis, and gene enrichment analysis (GSEA) were all used to show that STEAP1 is a potential prognostic biomarker for lung adenocarcinoma (LAUD) Guo et al. Feng and his team used Cox regression analysis and LASSO algorithm on a considerable number of DNA replication-related genes to stratify the postoperative tumor-free margin (R0) treated pancreatic ductal adenocarcinoma (PDAC) patients with different recurrence risks. This study resulted in identifying novel DNA replication-related gene signatures Feng et al. A combined approach using NGS data, karyotyping, mutation detection analysis, and flow cytometry was shown to be a better protocol for targeted therapy of acute myeloid leukemia (AML) patients El Omri et al. In summary, this research theme showed that data collected from genomic and bioinformatics analysis were effectively used to evaluate the prognostic value of biomarkers in cancer.

## Discussion

We had two aims with this RT: the first was to study genes and/or microRNAs that play a dual function in embryonic development and disease and to deliver an update about the applications of the genomics and bioinformatics advanced tools for understanding the complexity of the disease. To a large extent, these aims were met and achieved in the RT. All accepted articles were divided into 3 categories. The results published in the first category of the RT supported the notion of previous reports showing that many genes function in a dual role manner in development and disease (Gong et al., 2011; Teven et al., 2014; Sohn et al., 2016; Stepicheva and Song, 2016; Mahajan et al., 2021; McMillen et al., 2021; Assidi et al., 2022). Other reports showed that during development components of the FGF, Wnt, Notch, BPM, and Hedgehog signaling orchestrate crucial cellular events including proliferation, migration, differentiation, epithelial-mesenchymal transition (EMT), morphogenesis and somite myogenesis patterning. When gene expression controlling these events is dysregulated, it leads to disease induction (Hansson et al., 2004; Logan and Nusse, 2004; Ma et al., 2010; Teven et al., 2014; Xiao et al., 2017; Abreu de Oliveira et al., 2022; Alrefaei and Abu-Elmagd, 2022; Towler and Shore, 2022). Although our RT did not receive microRNA-related manuscripts, it is well-established that microRNAs play an important role in physiology and disease (Sayed and Abdellatif, 2011; Bhaskaran and Mohan, 2014; Kalayinia et al., 2021).

Stem cell medical research aiming at developing therapies has been recently the promise for chronic disease treatment (Mousaei Ghasroldasht et al., 2022). The current RT has contributed to this important line of research through three studies in which human BM-MSCs and Wharton’s Jelly cells were used to treat osteoarthritis and leukemia respectively Jafri et al., Kalamegam et al., and Huwaikem et al. In addition, stem cells can be also used for cell transplantation methods to cure diseases. For example, in this RT, Luo et al., showed successful CRISPR-mediated targeting of a Hemophilia A mutation, which may eventually one day be offered in the clinic as a treatment option.

For the second aim/category of the RT, a considerable number of published studies applied a wide range of genomic tools to unravel the causal gene(s) in disease Naseer et al., Naseer et al., Haque et al., Jung et al., Krämer et al., Zhan et al., Luo et al., Lv et al., Ma et al., Ouyang et al., and Özgümüs et al.

There was, in addition, substantial use in these studies of many advanced bioinformatics tools aimed at analyzing the involved molecular signaling pathways controlling the disease induction and progression Jafri et al., Kalamegam et al., and Huwaikem et al. In several studies, there was a good use of the freely available gene expression datasets for disease analysis Chen et al., Firneisz et al., Guo et al., Li et al., Qi et al., Yang et al., Feng et al., Yang et al., and Bahlas et al. However, the use of these data presents many ethical challenges (Lathe et al., 2008; Shabani and Borry, 2015). For the most part, these efforts intend to improve and identify early disease prediction, diagnosis, progression, and prognosis.

## Conclusion

Our Research Topic aimed at expanding our knowledge of the use of advanced genomic and bioinformatics tools. Results from several articles showed that genes could play a dual functional role in embryonic development and human disease. Stem cell-based therapy and CRISPR/cas9 mutation repair approaches are promising strategies for therapy. Several combined genomic approaches were successfully used to decipher disease induction mechanisms. Advances made in genomic and bioinformatic applications for understanding the complexities of human disease should be of great help to clinicians in personalized therapy.
